# Rebel honeybee workers have a tendency to become intraspecific reproductive parasites

**DOI:** 10.1002/ece3.4647

**Published:** 2018-10-31

**Authors:** Karolina Kuszewska, Krzysztof Miler, Wiktoria Rojek, Monika Ostap‐Chęć, Michal Woyciechowski

**Affiliations:** ^1^ Institute of Environmental Sciences Jagiellonian University Krakow Poland

**Keywords:** *Apis mellifera*, honeybee, intraspecific reproductive parasites, rebel workers, reproductive parasites

## Abstract

Worker honeybees may reproduce in either their own or foreign colonies; the latter situation is termed intraspecific reproductive parasitism (IRP). In this study, we compared the tendency for IRP between normal honeybee workers, which are characterized by a relatively low reproductive potential, and “rebel workers”, a recently discovered subcaste of honeybee workers characterized by a high reproductive potential that develops when the colony is without a queen. We expected that the high reproductive potential of the rebel workers would influence their reproductive strategy and that these individuals would drift to other colonies to lay eggs more often than normal workers. The results confirm our expectations and show that rebel workers are more likely than normal workers to drift to foreign colonies. The rebel workers also preferred to drift to queenless colonies than to queenright colonies, while the normal workers did not show this preference. This study indicates that rebel workers have a tendency for IRP, which may be responsible for the maintenance of the rebel worker strategy in bee populations.

## 1 INTRODUCTION

Some species are vulnerable to infiltration by cheater individuals that lay eggs to be reared in conspecific nests. Such intraspecific reproductive parasitism (IRP) is well known in birds (Yom‐Tov, 2001) and has also been described in a variety of insects, including the lace bug (*Necrophorus vespilloides*; Muller, Eggert, & Dressel, 1990) and an aphid (*Pemphigus obesinymphae*; Abbot, Withgott, & Moran, 2001). This behavior is also known in eusocial insects, for example, various species of bees (Beekman & Oldroyd, 2008; Lopez‐Vaamonde, Koning, Brown, Jordan, & Bourke, 2004; Nanork, Paar, Chapman, Wongsiri, & Oldroyd, 2005; Sumner, Lucas, Barker, & Isaac, 2007), ants (Heinze & Keller, 2000), and wasps (Klahn, 1988; Oliveira, Oi, Vollet‐Neto, & Wenseleers, 2016), and in these cases, workers with a higher than average reproductive potential drift to foreign nests to reproduce.

The honeybee (*Apis mellifera* L.) is a species in which individuals (workers) can drift between colonies; it was previously shown that, due to the often short distances between colonies in apiaries, up to 40% of the workers in a colony may be from other colonies (Pfeiffer & Crailsheim, 1998). It is believed that the drift of workers between colonies is more often caused by mistakes in perception and failures in orientation than by IRP (Pfeiffer & Crailsheim, 1998). These drifting individuals are usually sterile because the pheromones of the queen efficiently inhibit the development of ovaries in workers (Winston, 1987). However, in certain specific conditions, honeybee workers can activate their ovaries and lay unfertilized eggs that develop into males (drones). The most common factor that stimulates ovary activation and turn off the normal policing mechanisms that prevent workers from laying eggs (Ratnieks & Visscher, 1989; Woyciechowski & Lomnicki, 1987) is the absence of a queen and her pheromones in a nest (Winston, 1987). Therefore, workers that drift to a queenless colony may have a better chance of reproducing than workers that drift to a queenright colony. Indeed, this scenario was confirmed by previous research, which showed that workers that drift to queenless colonies have greater reproductive success than native individuals (Chapman, Beekman, & Oldroyd, 2010). The same research also showed that drifting workers had no preference between queenless or queenright colonies (Chapman et al., 2010), confirming that drift to foreign colonies is the result of mistakes in orientation. However, a different study showed that some individuals with activated ovaries tend to migrate to foreign colonies, with a preference for hopelessly queenless colonies (Yagound, Duncan, Chapman, & Oldroyd, 2017). Yagound et al. (2017) found that the probability to drift depends on both the subfamily of the worker (genetic aspect) and on the social environment, which directly influences the reproductive state of workers. Their study indicates that reproductive parasitism in honeybee workers is fairly common and that individuals with the opportunity to enter an unrelated colony often take advantage of it.

“Rebel worker” honeybees (Kuszewska & Woyciechowski, 2015; Kuszewska, Miler, Rojek, & Woyciechowski, 2017; Woyciechowski & Kuszewska, 2012) may shed some light on this question. Rebel workers are have higher reproductive potential because they have significantly more ovarioles in their ovaries than do normal workers. Other anatomical features differentiate the rebels from normal workers, including their more developed mandibular glands, which produce queen‐like pheromones, as well as underdeveloped hypopharyngeal glands, which suggests low production of brood food (Deseyn & Billen, 2005) and restricted nurse activity (Amdam et al., 2005). These anatomical differences show that rebel workers are more engaged than normal workers in laying their own male‐determined eggs. The proximate factor that influences rebel caste development is the absence of a queen or, more precisely, the lack of a queen's mandibular gland pheromones (Woyciechowski, Kuszewska, Pitorak, & Kierat, 2017) during the larval feeding period (unsealed larvae). If they remain in a queenless or a queenright colony during their adult lifetimes, these rebel workers display active ovaries (Woyciechowski & Kuszewska, 2012) and have a higher number of male offspring than normal workers (Kuszewska, Wącławska, & Woyciechowski, 2018). The appearance of workers with mature ovaries in orphaned colonies is not surprising as workers are known to lay eggs when a colony loses its queen and there is no chance of rearing a new queen (Page & Robinson, 1994; Velthuis, 1970). However, the readiness of rebel workers to reproduce in queenright colonies is more unexpected because of the hypothesis that the presence of a queen effectively inhibits oogenesis in workers (Page & Robinson, 1994; Velthuis, 1970), suggesting that their life strategy is highly tuned toward their own reproduction. The evolutionary explanation for this reproductive strategy in workers arises from the assumption of inclusive fitness theory (Hamilton, 1964), also known as kin selection theory (Maynard Smith, 1964), which can explain the altruistic strategies (Gadagkar, 1996; Nonacs, 2011) of colony members as well as certain conflicts between individuals in a nest (Ratnieks & Visscher, 1989; Woyciechowski & Lomnicki, 1987). Rebel workers develop because there is no queen in the nest during their larval stage, but the dramatic drop in relatedness between older‐generation workers and the offspring of a new queen after swarming seems to be the ultimate factor justifying the evolution and maintenance of this reproductive strategy (Woyciechowski & Kuszewska, 2012).

The increased reproductive potential and higher possibility of having activated ovarioles, even in queenright colonies, may influence the behavior of rebel workers and increase the possibility that they will drift to foreign colonies to lay their unfertilized eggs. To better understand these aspects of social behavior, we investigated whether rebel workers truly enter foreign colonies more often than normal workers of the same age, and we also determined how ovary development and activation influence the parasitic behavior of bees.

## 2 METHODS

### 2.1 Bee samples

The research was conducted in May and June 2017 in the experimental apiary of the Institute of Environmental Sciences (Jagiellonian University, Krakow, southern Poland). Four queenright honeybee (*Apis mellifera carnica*) colonies were studied, each consisting of 20,000–40,000 workers. All colonies were treated the same, and the experiment was conducted over four successive days. The experiment consisted of two stages and began when a queen was confined in two experimental frames to produce eggs of a similar age (day 0). Three days later, each colony was divided into queenright and queenless subunits (with equal distributions of resources, honeybee broods, and returning foragers), each with one experimental frame (day 3). Once the worker cells in the experimental frames were sealed (day 12), the subunits were reunited, so the experimental broods were maintained under the same conditions during their prepupal and pupal stages. Twenty‐two days after the start of the experiment, the frames with the newly emerged workers reared as larvae under queenright or queenless conditions were inserted into an incubator in the laboratory (34°C, 90% RH), and all workers that emerged within 24 hr were used in the experiment. The thoraxes of the workers from all colonies and groups were marked with a spot of paint Marabue Briliant Painter using different colors for different colonies, and all the workers were returned to their native hives. When the workers were 15 days old (before they began foraging), all the colonies in the apiary were carefully examined comb‐by‐comb, and all marked workers (normal and rebels) were collected. The collected workers were counted and killed by freezing to assess ovary development and activation. Additionally, whether the worker was collected in a native or foreign colony and the social status of the colonies (whether the given colony was queenless or queenright) were noted.

### 2.2 Examination of ovaries

The ovaries of the frozen workers were dissected and examined under a stereomicroscope. The ovarioles in both ovaries were counted followed by an assessment of ovary development using similar methods to those of previous studies (e.g., Woyciechowski & Kuszewska, 2012; Kuszewska et al., 2017). For analysis of ovary development, the most developed ovariole from each ovary was selected, and the maximum diameters of these two ovarioles (maximum width) were measured as described by Nakaoka, Takeuchi, and Kubo (2008), who reported that ovariole diameter accurately reflects ovarian activity. All ovaries were stained with Giemsa reagent (for approximately 10 s) before measurement.

### 2.3 Statistical analysis

**Figure 1:**
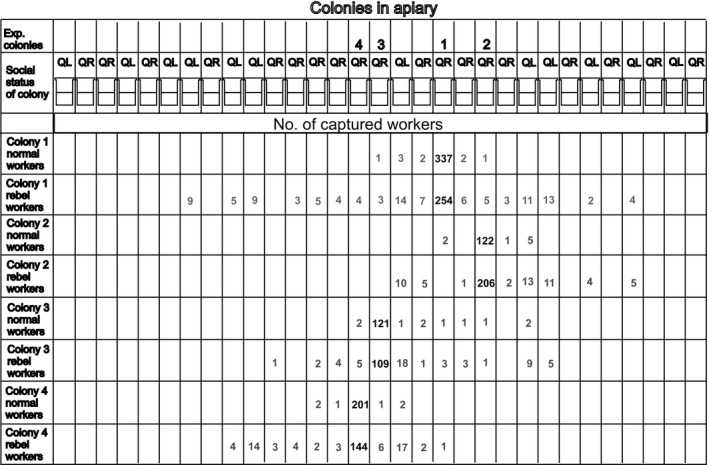
Apiary scheme showing the distribution of colonies (31 colonies including experimental colonies) and their social status (QR—queenright; QL—queenless). The scheme includes information about experimental colonies and where they were located in the apiary as well as information about the number of normal and rebel workers captured in their native colony (black number) or in foreign colonies (gray number)

The drifting behavior of normal and rebel workers was examined for each colony individually by Fisher's exact test, which compared the numbers of normal and rebel workers captured in both native and foreign colonies. To determine how often the bees drifted to a colony with or without a queen, the Mann–Whitney *U* nonparametric test was conducted for both the normal and rebel workers, but the number of bees collected in the queenless or queenright colonies was first divided by the total number of queenless or queenright colonies in the apiary, respectively (there were 19 colonies with a queen and 12 colonies without; Figure 1). This was done to overcome the error related to the different number of queenright and queenless colonies in the apiary.

To compare the number of ovarioles between the rebel and normal workers, a mixed‐model three‐way ANOVA was used with the type of worker (rebel vs. normal) and the type of target colony (native vs. foreign queenright vs. foreign queenless) as the fixed effects and the colony of origin as the random effect. However, the results indicated non‐significance of the random effect (*F*_3,247_ = 0.683; *p* = 0.6239), so the analysis was repeated with the random effect (the colony of origin) excluded (the results of this final model are shown). Statistically significant ANOVA results were followed by multiple comparisons using the post hoc Tukey HSD test, with *p* < 0.05 considered significant.

The differences in ovary activation between the normal and rebel workers were analyzed using the nonparametric Kruskal–Wallis test. Initially, whether there are differences in ovary activation between workers from different colonies of origin was determined. The six statistical tests showed that colony of origin was not an important factor, and we excluded this factor from subsequent analyses (Kruskal–Wallis tests: normal workers collected from native colony: *p* = 0.9232; rebel workers collected from native colony: *p* = 0.5105; normal workers collected from foreign queenright colonies: *p* = 0.5466; rebel workers collected from foreign queenright colonies: *p* = 0.8973; normal workers collected from foreign queenless colonies: *p* = 0.1217; rebel workers collected from foreign queenless colonies: *p* = 0.8656). After the exclusion of colony of origin as a contributing factor, a multiple‐comparison Kruskal–Wallis test was performed, as in Kuszewska and Woyciechowski (2013). All calculations for all experiments were performed with STATISTICA 11.0 (StatSoft, Poland).

## 3 RESULTS

**Figure 2:**
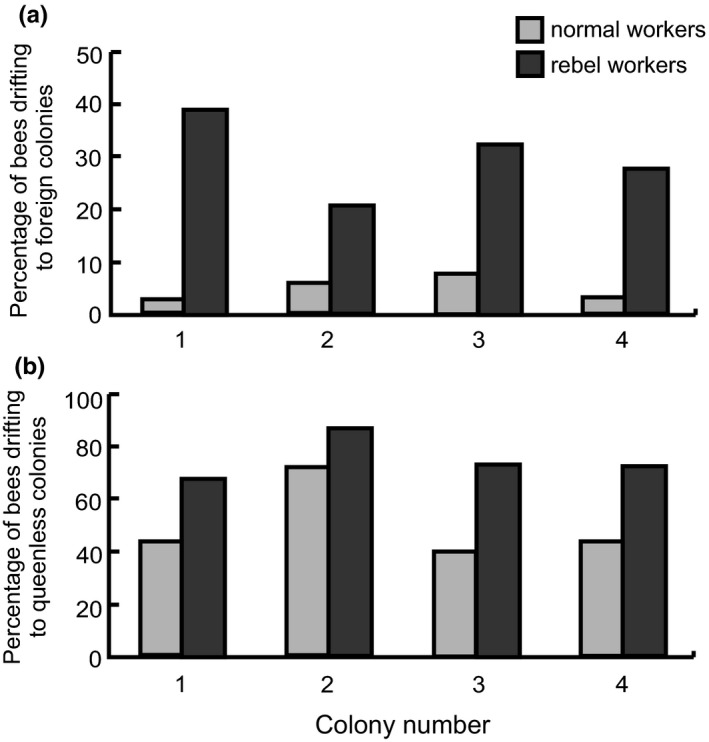
Percentage of normal workers (light gray bars) and rebel workers (dark gray bars) drifting from four experimental colonies to (a) foreign colonies (queenright and queenless colonies combined) and (b) queenless foreign colonies (including all drifting workers)

The number of colonies with or without queens and the number of bees collected from native or foreign colonies as well as information about the different colonies and groups are presented in Figure 1. The results showed that 20.5%–38.9% of the rebel workers drifted to foreign colonies while only 2.6%–7.6% of the normal workers did the same (Figure 2a). The difference in the proportion of rebel and normal workers drifting to foreign colonies was statistically significant for all tested colonies (Fisher's exact test; colony 1: *p* < 0.0001, colony 2: *p* = 0.0002, colony 3: *p* < 0.0001, colony 4: *p* < 0.0001). The rebel workers also preferred to drift to queenless colonies over queenright colonies (Mann–Whitney *U* test; *Z* = −2.125; *p* = 0.0304 Figure 2b), while the normal workers did not show a preference (Mann–Whitney *U* test; *Z* = −0.1452; *p* = 0.8845; Figure 2b).

**Figure 3:**
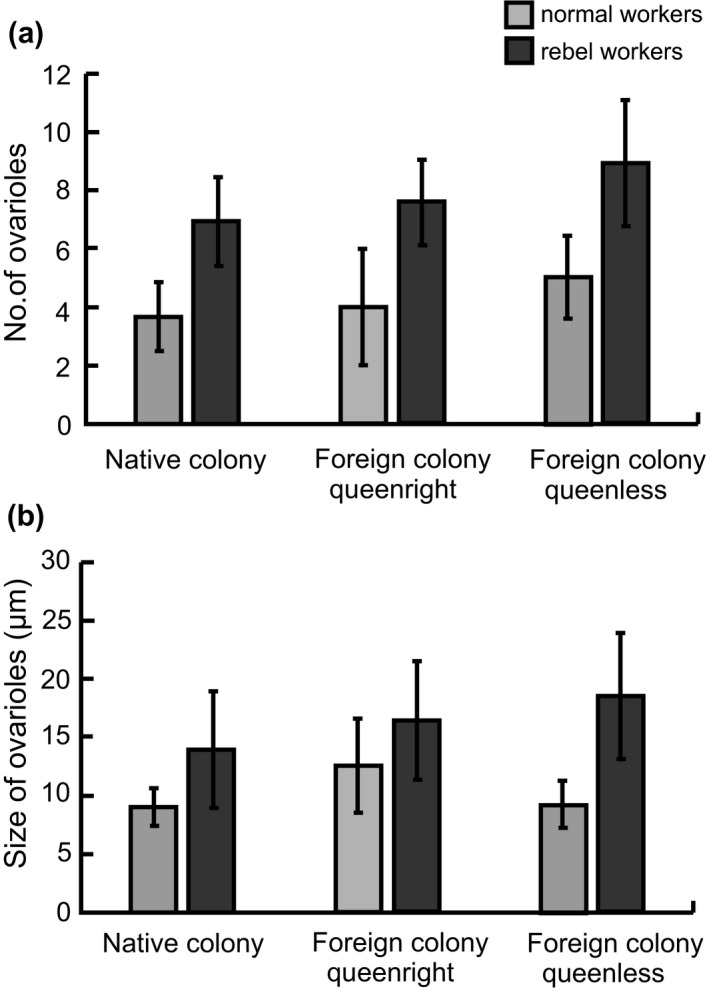
Anatomical parameters of normal workers (light gray bars) and rebel workers (dark gray bars) captured in native, foreign queenright, and foreign queenless colonies; (a) the number of ovarioles (means ± *SD*) and (b) ovariole size (means ± *SD*). Data were pooled from all colonies

This study also showed that rebel workers had more ovarioles than normal workers (two‐way ANOVA, the type of workers: *F*_1,265_ = 424.572; *p* < 0.0001; Figure 3a). The number of ovarioles also differed between workers caught in native or foreign colonies (two‐way ANOVA, the type of target colony: *F*_2,265_ = 17.559; *p* < 0.0001), and the interaction between the type of worker (rebel or normal) and the type of target colony was also significant (two‐way ANOVA, interaction of the type of worker and the type of target colony: *F*_2,265_ = 5.337; *p* = 0.0053). The post hoc test showed that the number of ovarioles in the normal workers remained the same among individuals collected from native or foreign queenright or queenless colonies (Tukey's test; *p* > 0.05; Figure 3a), while the rebel workers found in native colonies had fewer ovarioles (Tukey's test; *p* = 0.0015; Figure 3a) than those found in foreign queenright colonies (Tukey's test; *p* < 0.0001; Figure 3a) and foreign queenless colonies (Tukey's test; *p = *0.0027; Figure 3a), in which they had the highest number of ovarioles.

The rate of ovariole activation also depended on worker type; rebel workers had more activated ovarioles than normal workers (Kruskal–Wallis *p* < 0.0001). Ovary development did not differ among normal workers collected from different colony types (Kruskal–Wallis *p* = 1.0000; Figure 3b), but the rebel workers collected from foreign colonies without queens had better developed ovaries (Kruskal–Wallis *p* = 1.0000; Figure 3b) than the rebel workers collected from native colonies (Kruskal–Wallis *p* = 0.0006; Figure 3b). The rebel workers found in the foreign queenright colonies had an intermediate level of ovary development, but it did not differ from that of rebel workers from native (Kruskal–Wallis *p* = 1.0000; Figure 3b) and foreign queenless colonies (Kruskal–Wallis *p* = 1.3837; Figure 3b).

## 4 DISCUSSION

The results of our study are the first to show that rebel workers, individuals with a higher reproductive potential than normal workers, migrate to foreign colonies more often than normal workers. Two different hypotheses may explain this behavior. First, rebel workers migrate to foreign colonies more often because they have a greater likelihood of laying their own eggs and begin to behave as an intraspecific parasite. Second, the rebel workers' behavior can be explained as a result of mistakes in orientation rather than parasitic behavior. We tested these hypotheses by assessing the number of ovarioles and ovary activation among rebel workers collected from native, foreign queenright, and foreign queenless colonies. The distinction between ovary activation and number of ovarioles in the ovary was very important because these two parameters are determined in different periods of the worker's life. The number of ovarioles in the ovary is determined during the larval period and depends on the quality and quantity of food. The number of ovarioles usually does not change during adult life in honeybees. In contrast, ovary activation is determined during adult life in workers and depends on many factors, including the presence or absence of a queen and her pheromones, the presence of brood, and the task performed by the workers. These factors suggest that number of ovarioles can be a reason for a bee to migrate to other colonies, while ovary activation can be considered a result of this migration. This study showed that rebel workers that migrate to foreign colonies have a higher number of ovarioles in comparison to rebel workers that remain in their native colony. The same tendency was not observed among normal workers. Moreover, more detailed results showed that rebels with the highest number of ovarioles prefer to migrate to queenless colonies, where the likelihood of successful reproduction is higher than that in queenright colonies because of the absence of queen pheromones (Winston, 1987) and the ineffective policing system (Ratnieks, 1993). It is possible that rebels sense orphaned colonies and target them as places to lay eggs, and a higher number of ovarioles makes them more prone to seek such conducive locations. This study also showed that the ovaries of rebel workers were least activated in native colonies and most activated in queenless foreign colonies. The same tendency was not found among normal workers collected from different types of colonies. Higher ovary activation in a foreign colony was not surprising results because workers typically have a greater likelihood of ovary activation in a foreign as well as queenless colony (Miller & Ratnieks, 2001; Yagound et al., 2017). Additionally, some studies showed that ovary activation is related to the number of ovarioles (Makert, Paxton, & Hartfelder, 2006). Thus, the ovary activation in rebel workers collected from foreign colonies might have been higher because these individuals had more ovarioles in the ovary and spend life in alien colony, but we have no evidence to exclude the alternative scenario that rebel workers with more activated ovaries are more likely to drift to other colonies, as suggested by Yagound et al. (2017).

In contrast to the previously mentioned study (Yagound et al., 2017), we did not find more ovarioles and more activated ovaries in normal workers that drifted to foreign colonies compared to those that stayed in native colonies, but this result is easy to explain. Previous studies examined large numbers of normal workers and their tendency to migrate, while the total number of normal bees that drifted to other colonies was very low in our experiment (a maximum of 10 individuals in every colony), and this number was probably not sufficiently large to statistically determine the reproductive differences between bees in native and foreign nests, even if this tendency does exist in honeybee colonies.

One of the objections to the present research may be the spatial location of honeybee nests in the apiary because the nests are arranged in rows, as illustrated in Figure 1, and workers usually prefer to migrate to the closest colonies (Figure 1). The same tendency was also described previously (Yagound et al., 2017). It is possible that such spatial arrangement of nests in the apiary can influence bees behavior because the workers can randomly migrate to foreign colonies that are near the experimental colony, regardless of whether they are colonies with or without queens. However, we think that this orientation did not have a significant impact on the results of this study and their interpretation. Firstly, this study compared the tendency to migrate to foreign colonies between two different groups—normal and rebel workers—coming from the same colony. The rebel workers more often migrated to other colonies, regardless of the reason for the migration. Secondly, the experimental colonies were usually in the neighborhood of queenright colonies (with exception of Colony 3, Figure 1) and despite this condition, more rebel workers preferred to migrate to queenless colonies.

We also cannot completely exclude the possibility that rebel workers are more often found in foreign colonies because they drift between colonies due to mistakes in perception and failures in orientation (Chapman et al., 2010; Pfeiffer & Crailsheim, 1998; Smith & Loope, 2016). The investment in reproduction (Mery & Kawecki, 2003) as well as in certain learning abilities is costly (Miler, Kuszewska, Zuber, & Woyciechowski, 2018) thus, there may exist some trade‐off between these two physiologically traits. Indeed, a study investigating the fruit fly (*Drosophila melanogaster*; Mery & Kawecki, 2003) showed that individuals from a line selected for “low learning” oviposited more eggs than individuals from a line selected for “high learning,” indicating that a trade‐off may exist between the learning ability and reproductive potential of animals and that individuals with a higher reproductive potential may be characterized by lower learning skills. However, in our opinion, this trade‐off between learning and reproductive potential cannot sufficiently explain the results of our study. If rebel workers were stupider than normal workers, we would have caught the same number of lost bees from queenright and queenless foreign colonies, but our results indicate completely opposite scenarios. There is no reason that disoriented bees should tend to choose a new type of colony. Regardless of whether rebel workers drift to other colonies because their learning and spatial skills are lower than those of normal workers or whether they specifically target foreign colonies to cheat those members, they show increased intraspecific parasitism.

As noted in the introduction, the appearance of rebel workers in honeybee colonies was predicted from assumptions of inclusive fitness theory (Hamilton, 1964), also known as kin selection theory (Maynard Smith, 1964); the tendency of rebels to be reproductive parasites is also consistent with this theory. Rebel workers with activated ovaries that drift to other colonies behave altruistically to their own colony members; their relatives do not bear the cost of caring for their offspring nor do they use the resources of their native colony. Moreover, this cheating behavior by rebel workers probably contributes to the maintenance of this caste in bee populations due to the reproductive benefits of this strategy.

Our study showed that rebel workers are more likely to drift to other colonies and function as reproductive parasites than normal workers. This behavior is consistent with kin selection theory because rebels should use the resources of unrelated colonies to raise their own male offspring rather than those of their own colony. Apparently, rebel workers not only target unrelated colonies but also prefer those that are queenless, which plausibly increases the chances of their sons being reared.

## CONFLICT OF INTEREST

The authors declare no conflict of interest.

## AUTHOR CONTRIBUTIONS

Conceived of and designed the experiments: KK. Performed the experiments: KK, KM, WR, and MOC. Analyzed the data: KK. Contributed reagents/materials/analysis tools: KK, KM, and MW. Wrote the paper: KK, MW, KM, WR, and MOC.

## DATA ACCESSIBILITY

All original data are included with the manuscript submission, either in the manuscript (Figure 1) or as an additional Excel file.
